# Single cardiac ventricular myosins are autonomous motors

**DOI:** 10.1098/rsob.170240

**Published:** 2018-04-18

**Authors:** Yihua Wang, Chen-Ching Yuan, Katarzyna Kazmierczak, Danuta Szczesna-Cordary, Thomas P. Burghardt

**Affiliations:** 1 Department of Biochemistry and Molecular Biology, Mayo Clinic Rochester, 200 First Street SW, Rochester, MN 55905, USA; 2 Molecular and Cellular Pharmacology, University of Miami Miller School of Medicine, Miami, FL 33136, USA; 3 Department of Physiology and Biomedical Engineering, Mayo Clinic Rochester, Rochester, MN 55905, USA

**Keywords:** single cardiac myosin mechanics, super-resolution microscopy, ratcheting myosin essential light chain, Qdot labelled actin under load, cardiomyopathy-linked mutants

## Abstract

Myosin transduces ATP free energy into mechanical work in muscle. Cardiac muscle has dynamically wide-ranging power demands on the motor as the muscle changes modes in a heartbeat from relaxation, via auxotonic shortening, to isometric contraction. The cardiac power output modulation mechanism is explored *in vitro* by assessing single cardiac myosin step-size selection versus load. Transgenic mice express human ventricular essential light chain (ELC) in wild- type (WT), or hypertrophic cardiomyopathy-linked mutant forms, A57G or E143K, in a background of mouse α-cardiac myosin heavy chain. Ensemble motility and single myosin mechanical characteristics are consistent with an A57G that impairs ELC N-terminus actin binding and an E143K that impairs lever-arm stability, while both species down-shift average step-size with increasing load. Cardiac myosin *in vivo* down-shifts velocity/force ratio with increasing load by changed unitary step-size selections. Here, the loaded *in vitro* single myosin assay indicates quantitative complementarity with the *in vivo* mechanism. Both have two embedded regulatory transitions, one inhibiting ADP release and a second novel mechanism inhibiting actin detachment via strain on the actin-bound ELC N-terminus. Competing regulators filter unitary step-size selection to control force-velocity modulation without myosin integration into muscle. Cardiac myosin is muscle in a molecule.

## 1. Introduction

The myosin motor protein powers muscle contraction with chemomechanical transduction of ATP free energy into the mechanical work of actin translation against resisting force [1]. Skeletal and cardiac muscle have actomyosin interacting cyclically and mostly stochastically generating mechanical energy from ATP hydrolysis under conditions demanding dynamically wide-ranging power [2]. Muscle myosin has a motor domain transducer containing ATP and actin-binding sites, and a mechanical coupler linking impulses from the actin-bound motor to the myosin thick filament. The mechanical coupler is a lever arm stabilized by bound essential and regulatory light chains (ELC and RLC) that rotates cyclically to impel bound actin [3,4]. Linear actin displacement due to lever arm rotation is the myosin unitary step-size [5] that partitions into sub-steps [6,7]. We showed in earlier work using the Qdot *in vitro* motility assay that porcine cardiac ventricular myosin (*β*mys) has three distinct unitary step-sizes of approximately 3, 5 and 8 nm that move actin in the absence of load with approximately 15, 50 and 35% relative step-size frequencies, respectively [8]. The cardiac-specific N-terminal extension of myosin ELC mediates the step-size frequency modulation [9] supporting a substantial velocity dynamic range [10]. In addition to cardiac myosins, the three-step-size mechanism appears in other muscle myosins including zebrafish skeletal [11,12].

The complexity and density of the muscle sarcomere prohibited sensing single myosin *in vivo* until the visible light transparent zebrafish embryo opened a window into time-resolved single myosin mechanics. The method imaged rotation or tilt of a single myosin lever arm domain tagged with photoactivatable GFP in transgenic zebrafish embryo skeletal and cardiac muscle as it converts motor generated torque into the linear unitary step [11,12]. The *in vivo* single myosin data showed that cardiac myosin has three distinct (slightly foreshortened due to strain [13]) unitary step-sizes of approximately 2, 4 and 6 nm moving actin with relative step-size frequencies that change dramatically as the muscle changes from auxotonic to near-isometric contraction. We concluded that cardiac muscle *in vivo* regulates force–velocity by remixing the three different myosin unitary step-sizes with changing step-size frequencies and proposed that the ELC N-terminus is a strain sensor competing with the traditional lever arm strain sensor [14] to modulate step-frequency. The proposed ELC N-terminus strain sensor has a ratchet-like selective resistance to movement in the direction of the loading force, while permissive to movement in the direction of the contractile force.

*In vivo* myosin cross-bridges function within a structured environment of the sarcomere where densely packed myosin thick filaments impel complementary actin thin filaments in a lattice also occupied by proximal ancillary proteins. Density, proximity and complexity in the *in vivo* environment imply a potential for hierarchical coordinated ensemble myosin functionality. *In vitro*, purified, isolated and independent myosins also generate mechanical power by translating actin against resisting force. Whether unitary step-size selection for force–velocity regulation is a systemic property of the sarcomere or an intrinsic property of an autonomous myosin is tested by comparing the step-size and step-frequency signatures for both *in vivo* and *in vitro* venues. Here, we focus on the *in vitro* measurements. In the present study, we measure motility velocity, step-size and step-frequency of myosin under constant loads. These data fulfil requirements to measure force–velocity for single and ensemble myosins *in vitro*.

We investigated the effect of load *in vitro* on three transgenic mouse cardiac myosins WT, A57G and E143K. The control WT myosin has the native human cardiac ELC (MYL3) replacing mouse cardiac ELC, and the mutants have the human cardiac ELC with the A57G or E143K substitutions. The hypertrophic cardiomyopathy (HCM)-linked ELC mutant A57G [15] in a transgenic mouse model indicated higher myofilament calcium sensitivity, probably causing pathological cardiac remodelling and increased cardiac output and stroke work [16]. In the human heart, E143K causes HCM with restrictive physiology [17]. The transgenic mouse model for E143K has cardiomyopathy with abnormalities in diastolic function, fibrosis, and reduced cardiac output and stroke work [18]. We find here that evident differences *in vitro* among the WT and mutant myosins reflect principally the impact of A57G on the ELC-ratchet strain sensor and the impact of E143K on lever arm stiffness.

Characteristics common to the *in vitro* human cardiac myosin models from mouse and native *in vivo* zebrafish cardiac myosin species are compelling quantitative indicators for myosin autonomy. The WT and mutant myosins studied here, like their native *in vivo* counterpart in zebrafish [12], down-shift ensemble displacement under load. Step-size shifting is the adapted average step-size done by affecting step-frequency, step-size or both, in the unitary steps. Thus, the ensemble recombination of step-sizes appropriate for specific loading conditions is managed by individual heads and does not require motor integration into whole muscle tissue. These results extend the longstanding observation of myosin autonomy in a muscle [19] to individual motors.

## 2. Material and methods

### 2.1. Protein preparations

Cardiac myosin was isolated from groups of mouse hearts—WT (14 hearts), A57G (14 hearts) and E143K (6 hearts)—according to Kazmierczak *et al*. [20]. Whole hearts were isolated, the atria removed and ventricles (left and right) were flash-frozen immediately and stored at −80°C until processed. The ventricular tissue was later thawed in ice-cold Guba Straub-type buffer, pH 6.5 consisting of 300 mM NaCl, 100 mM NaH_2_PO_4,_ 50 mM Na_2_HPO_4,_ 1 mM MgCl_2,_ 10 mM EDTA, 0.1% NaN_3_, 10 mM Na_4_P_2_O_7_, 1 mM DTT and protease inhibitor cocktail in a volume of 0.75 ml buffer per 0.2 g tissue. Ventricles kept on ice were first minced by hand and then homogenized for 2 min at 30 Hz in a Mixer-mill MM301 (Retsch). The homogenate was then incubated on ice for 40 min before centrifugation at 200 000*g* for 1 h. The supernatant was then diluted 60-fold with ice-cold water with 2 mM DTT and incubated on ice for 30 min with stirring and left standing for an additional 30 min. The samples were centrifuged again at 8000*g* for 10 min and resultant pellets were then re-suspended in minimal volume of buffer containing 0.4 M KCl, 10 mM MOPS, pH 7.0, 5 mM DTT and protease inhibitor cocktail. Samples were then diluted 1 : 1 with glycerol, mixed gently and were shipped on ice the same day of the prep from Miami to Rochester. Proteins were stored at −20°C until used for experiments within 12 days of arrival.

### 2.2. Actin-activated myosin ATPase

Mouse cardiac ventricular myosin (*α*mys) stored in 50% glycerol was precipitated with the addition of 12 volumes of ice-cold water containing 2 mM DTT, collected by centrifugation, and then resuspended in 300 mM KCl, 25 mM imidazole (pH 7.4), 4 mM MgCl_2_, 1 mM EGTA, 10 mM DTT and 10 µg ml^−1^ leupeptin. Myosin at a final concentration of 0.6 µM was titrated with 1, 3, 5, 9, 22, 50 and 93 µM actin. The ATPase assay buffer contained 25 mM imidazole (pH 7.4), 4 mM MgCl_2_, 1 mM EGTA, 10 mM DTT, 10 µg ml^−1^ leupeptin and a final KCl concentration of 25 mM. ATPase reaction was initiated by the addition of 3 mM ATP, and the mixture was incubated at 21°C for 5 min. Inorganic phosphate measurements were performed using the Fiske & Subbarow method [21].

Actin-activated ATPase data were fitted using Michaelis–Menten kinetics,2.1
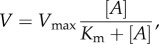
for actin concentration [*A*], *V* the ATPase rate equal to *V*_max_ at saturating actin and actin-binding constant *K*_m_. *V* versus [*A*] curves were fitted using a nonlinear algorithm to determine constants *V*_max_ and *K*_m_.

### 2.3. *In vitro* motility and Qdot assays

*In vitro* motility and Qdot assays were performed in a flow cell using total internal reflection fluorescence (TIRF) microscopy exactly as described in [9]. Motility buffer included 25 mM KCl, 25 mM imidazole (pH 7.4), 4 mM MgCl_2_, 1 mM EGTA, 20 mM DTT, 10 µg ml^−1^ leupeptin, 0.7% methylcellulose, 2 mM ATP, 3 mg ml^−1^ glucose, 0.018 mg ml^−1^ catalase and 0.1 mg ml^−1^ glucose oxidase. The flow cell was infused at the start with 0.04–0.4 µM myosin. Actin sliding velocities for the *in vitro* motility assay, *s*_m_, and the length of actin filaments were quantitated using FIESTA software [22]. Peak velocities were obtained with a minimum of approximately 0.16 µM myosin for each variant.

Frictional loading assays were performed like the unloaded assays except that the flow cell was infused at the start with the mixture of myosin and α-actinin in concentrations of 0.16 µM myosin and 0–6 µg ml^−1^ α-actinin (Cytoskeleton, Denver, CO).

In the Qdot assay, images were acquired with an EMCCD camera (Andor, Belfast, UK) in 45 ms intervals indicated by Δt and using Andor's SOLIS software. Each movie was recorded for 36 s. Intensity values were converted to photons using the conversion formula in SOLIS and the images output in TIFF format for reading into ImageJ. We tracked the movement of the Qdot labelled actin at super resolution using the ImageJ plugin QuickPALM [23]. Baseline histograms corresponding to thermal/mechanical fluctuations were recorded likewise from Qdot labelled actin immobilized on the surface by myosin in the absence of ATP. Thermal/mechanical fluctuation baseline contributions to the event–velocity histograms were estimated as described below (see ‘Qdot assay event–velocity histogram simulation). All *in vitro* motility and Qdot assay experiments were conducted at room temperature (20–22°C).

### 2.4. Nanoscale measurement calibration

Calibration demonstrates a robust reliability for measuring nanometre-size steps as described previously [9]. Briefly, Qdots adsorbed to a glass slide were observed using TIRF as in the motility assay. A nanopositioning stage (Mad City Labs, Madison, WI) translated the slide under Labview (National Instruments, Austin, TX) control performing steps randomly selected from the {3, 5, 8} nm set and with step-frequencies {0.125, 0.5, 0.375} imitating cardiac myosin behaviour. Qdot assay velocity quantitation for calibration is identical to that used for Qdot labelled actin under myosin-driven motility. Results indicated the assay accurately reflected the nanometre scale movement (see Qdot assay calibration and compliance in results of Wang *et al*. [9]).

### 2.5. Authentication

We authenticate the Qdot assay by identifying the nature and origin of the single actomyosin interactions detected as described previously [9]. We documented heterogeneous actin filament velocity as it moved over the myosin fixed to the coverslip. The unloaded filaments frequently encounter low surface density myosin causing 1%–3% of the displacements measured to be from single actomyosin impulses (see [9]). The evidence is similar for the loaded filaments in this study except that under load we expect myosin detachment rate to decrease to lower the fraction of unitary steps observed in the event–velocity histogram. We find the thermal/mechanical fluctuation baselines are a more dominant feature of the raw data in agreement with expectations. Nonetheless, the characteristic unitary impulsion is readily identified and quantitated.

### 2.6. Force calibration in the loaded actin *in vitro* motility and Qdot assays

Loaded *in vitro* motility and Qdot assay data were fitted to a viscoelastic model of frictional loads as suggested by Greenberg & Moore [24]. Average sliding filament velocity, *s*_m_, is given by2.2
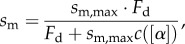
for *s*_m,max_ the velocity at zero load, *F*_d_ the ensemble myosin driving force and friction coefficient *c*([*α*]) given by2.3
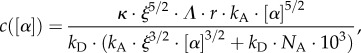
for *κ* system compliance associated with α-actinin (1.7 pN nm^−1^), *ξ* a constant defining the surface geometry of α-actinin on the flow cell surface (3.97 × 10^21^ M^−1^ m^−2^), *Λ* actin filament length, *r* the reach of α-actinin to bind to the actin filament (82 nm), *k*_A_ the second-order rate constant for α-actinin attachment to the actin filament (4 × 10^6^ M^−1^ s^−1^), [*α*] molar concentration of α-actinin, *k*_D_ α-actinin detachment rate (9.6 s^−1^) and *N*_A_ Avogadro's number. *s*_m,max_ and *F*_d_ were estimated by fitting velocity data from the loaded assay using nonlinear least squares fitting in Matlab (The MathWorks, Natick, MA).

The frictional force, *F*_f_, exerted by α-actinin in the loaded assay is given by2.4
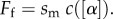


Values for parameters used in equations (2.2) and (2.3) are identical to those already described [24] except for fitted parameters *s*_m,max_, *F*_d_, and the length of actin filament, *Λ* ≈ 1 µm in this work. Constants in equations (2.2) and (2.3) (except *N*_A_) are estimates. They introduce errors affecting absolute but not relative force–velocity estimates. Results shown subsequently reflect random errors suitable for comparing myosins. Frictional force–velocity curves were fitted to the Hill equation [25] given by2.5
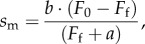
where *F*_0_ is the isometric force, *a* = 210 pN and *b* = 0.4 µm s^−1^. *F*_0_, *a* and *b* are fitted parameters with *F*_0_ different for each species, while *a* and *b* are constant for all species. Power output *P* is given by2.6
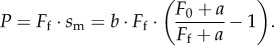


The maximal power output occurs at the point where the derivative of the power with respect to the load, *F*_f_, is equal to zero. The error in the power output and the load at maximal power were calculated by propagating the error from the nonlinear least squares fitting.

### 2.7. Qdot assay event–velocity histogram simulation

*In vitro* motility has the myosin moving actin with a motility velocity *s*_m_ such that2.7
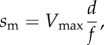
for myosin unitary step-size *d* and duty-ratio *f* [26]. Duty-ratio is the time actomyosin is strongly bound during an ATPase cycle, *t*_on_, divided by the cycle time, 1/*V*_max_, hence2.8
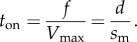


Myosin executes one of three unitary steps with step-size, *d_j_*, and relative step-frequency, *ω_j_*, for unitary step *j* *=* *S, I* and *L* where *S*, *Ι* and L are for the short (approx. 3 nm), intermediate (approx. 5 nm) and long (approx. 8 nm) nominal unitary steps of cardiac myosin [8,27]. Relative step-frequency is a characteristic proportional to the rate of cross-bridge cycling with the higher rate producing a more frequent *j*^th^ step with step-size *d_j_*. The dimensionless relative step-frequency is normalized with *ω*_S_ + *ω_Ι_* + *ω*_L_ = 1. The absolute cycling rate for step *j*, *V_j_*, has *V_j_* = *V*_max_ *ω_j_* and with 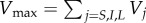.

In an ensemble of cross-bridges interacting with one actin filament, like the conditions in every muscle or motility assay, only one actin velocity is possible; hence, motility velocity *s*_m_ is the same for each unitary step-size implying each step-size has unique duty ratio and time strongly actin bound. From equation (2.8), step *j* duty ratio2.9
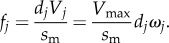


From equations (2.8) and (2.9), the time myosin spends strongly bound to actin2.10
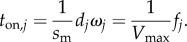


Equation (2.10) shows that each unitary step has a unique *t*_on_ that varies with step-size and relative step-frequency. *t*_on_ is distributed exponentially as observed using the laser trap for the *in vitro* actin detachment rate [28]. Ensemble average quantities used include average step-size2.11
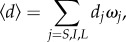
average duty-ratio 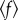, time strongly actin bound 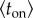
2.12
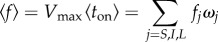
and average myosin power output from equation (2.6).

Simultaneously bound cross-bridges produce identical actin sliding velocity, *s*_m_, for step-size, *d*, and the time the cross-bridge is actin bound, *t*_on_, such that *s*_m_ = *d*/*t*_on_. Net velocity is proportional to the time any cross-bridge is actin bound between frames. At higher velocities, when multiple cross-bridges impel actin, it is more likely that two or more will overlap some of their bound time, producing net sliding velocity intermediate to the discrete velocities even within the domain of the unitary step-size velocity. This effect is seen as the rising baseline in the panels that becomes more significant at higher resisting force with rising 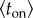 due to strain.

We simulated motility assay event–velocity histograms using a time × space array representing an actin filament interacting with the surface-bound myosin. Array rows represent myosin-binding sites on actin located every 36 nm along the filament that interacts with surface-bound myosins, while columns represent their time evolution. We simulated an approximately 1 µm long actin filament having 28 total myosin-binding sites. The time × space array was filled one row at a time by randomly generating binding site occupation from actin-binding probability *p*.

We partitioned *t*_on_ into *n* segments (*t*_seg_ = *t*_on_/*n*). An occupied binding site remains occupied for several *t*_seg_'s (one *t*_seg_ elapses between rows in the time × space array) and on average for 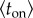. The total array column length is equal to Δ*t*/*t*_seg_, for Δ*t*, the time interval between consecutive frames. The actin filament velocity is then the number of rows with at least one site occupied times the myosin step-size divided by nΔt. The simulation for a single actin filament and for Δ*t* = 45 ms is run repeatedly until the number of events falling within the unitary step-size domain matches the number observed.

Experimental, **v**_obs_, and simulated data, **v**_sim_, are event–velocity histograms in a vector representation. Baseline histograms corresponding to thermal/mechanical fluctuations measured from immobilized Qdots, **v**_t/m_, were fit to **v**_obs_, using the relationship **v**_obs_ = **v**_sim_ + c **v**_t/m_, where c is the unknown scalar. Constant c is estimated by least-squares minimization with c = **v**_t/m_^T^(**v**_obs_ − **v**_sim_)/(**v**_t/m_^T^·**v**_t/m_) where ^T^ means transpose.

V_max_ and motility velocity, s_m_, are measured under saturating actin and myosin conditions, respectively. They are constant parameter inputs to the simulation that are characteristic to each myosin tested. Simulation approximates the Qdot motility event–velocity histogram in the low velocity domain of 0–4 natural velocity units (vu) where 1 vu = (*d*_I_/Δ*t*) for *d*_I_ the intermediate step-size (near 5 nm) and where unitary events dominate. The unknown parameter set actively searched in the simulation consists of the actin-binding probability for myosin *p* (1 free parameter), step-size (3 free parameters) and relative step-frequency (3–1 = 2 free parameters due to normalization). Trial parameter values are generated in the simulation by random choice from a range of values set at the start of the simulation as described below. Simulations sampled all of parameter space. Peak position and area for the first three peaks in event–velocity histograms are the unitary step-size and relative step-frequency estimates obtained directly from inspection of the data. We observed three step-sizes each distributed normally and with standard deviations (SDs) within 0.3–0.7 nm, and three step-frequencies each distributed normally and SD within 0.1–0.3. We searched step-size and step-frequency parameter space in five dimensions over a range defined by ±1.5 s.d. in seven intervals. The actin-binding probability for myosin and its parameter space dimensions were estimated empirically and spanned in seven intervals during the search for best fitting simulations. Estimates for step-size and step-frequency from simulation rigorously account for step overlap when two or more myosins impinge on one actin filament during some of the time they are strongly actin bound.

A Qdot assay dataset consists of 8–19 acquisitions (one acquisition is one *in vitro* motility movie and corresponding event–velocity histogram) from preparations of WT, A57G and E143K. Two or three separate protein preparations were used, giving a total of 16–55 acquisitions for each protein at each actin loading. Comparison of simulated curves to data uses the *χ*^2^ goodness-of-fit test that is weighted by event total then summed over all the acquisitions for evaluating global goodness of fit.

Simulated data ensembles were created by using the 16–21 best-fitting event–velocity histogram simulations generated for a Qdot assay dataset. The simulations are combined linearly to approximate the measured event–velocity histogram from the pooled data (when appropriate, see ‘Statistics’ below) with coefficients ≥0 while minimizing the *χ*^2^ goodness-of-fit test with all points equally weighted.

### 2.8. α-actinin attachment/detachment effect on myosin step-size

The attachment/detachment of α-actinin to actin does not actively produce actin movement implying its possible contribution to the myosin step-size will be stochastic (unlike myosin with step-size constrained by lever arm dimensions). Average α-actinin ‘step-size’ is equal to the time α-actinin spends detached from actin (*τ*_off_) times average actin motility velocity. The α-actinin actin-binding rate, 1/*τ*_off_ = *k*_A_ {*ξ*[*α*]}^3/2^ 10^−3^ N_A_^−1^ s^−1^ for *k*_A_ = 4 × 10^6^ M^−1^ s^−1^, *ξ* = 3.97 × 10^21^ M^−1^ m^−2^, [*α*] the α-actinin bulk concentration in M, N_A_ Avogadro's number and 10^−3^ needed to convert m^3^ to litres. When [*α*] = 5 µg ml^−1^ (=0.05 µM), 1/*τ*_off_ ≈ 18.6 s^−1^ corresponding to *τ*_off_ of approximately 54 ms. Average Qdot velocity for this condition is approximately 0.6 µm s^−1^ giving average displacement of 32 nm far beyond our estimates for the myosin step-size. Lower [*α*] produces a longer average displacement. Furthermore, we observe constant step-sizes and no apparent dependence on ensemble motility velocity consistent with their dependence on myosin dimensions. The α-actinin attachment/detachment to actin does not affect the estimate of myosin step-size.

### 2.9. Fraction of actin filaments under isometric load

Actin filaments under load produce a myosin state characterized by the zero step-size with step frequency, *ω*_0_, measuring the fraction of all myosins in isometric contraction. The *ω*_0_ is estimated from loaded motility velocity, *s*_m_, such that2.13
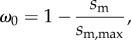
where *s*_m,max_ is the velocity in the absence of load. We define new step frequency fractions, *x*_0_, *x*_S_, *x*_I_ and *x*_L_ that sum to 1 and such that2.14
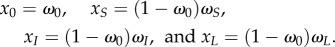


We use them when computing myosin flux through the pathways producing the various step-sizes as discussed subsequently.

### 2.10. Statistics

*In vitro* motility experiments using WT, A57G and E143K preparations corresponded to 16–55 independent event–velocity histograms for each protein. We simulated data from each protein preparation independently to estimate single myosin mechanical characteristics consisting of three step-sizes and three step-frequencies. We compared three step-sizes or 3 step-frequencies as categorical variables in factor 1 and the 2–3 independent datasets acquired for the separate protein preparations (with 8–19 independent event–velocity histograms for each species) in factor 2 using two-way ANOVA with Bonferroni or Tukey–Kramer post-tests for significance at the 0.05 level. This test indicated no significant difference among the independent datasets for each species hence datasets were pooled for a given protein.

### 2.11. Quantitative effect of myosin mixtures

Methods up to this point pertain to homogeneous protein samples containing a single myosin species. Below we discuss quantitating characteristics of a heterogeneous myosin sample. We describe here specific material properties of the myosins that can depend linearly or nonlinearly on their relative content. HCM-linked mutations *in vivo* have dominant negative effects largely independent of the percent of protein expression [18].

The adult mouse ventriculum contains principally the MYH6 heavy chain gene expression product with ≤ 1% MYH7 expression. This is true for both nontransgenic (NTg) and transgenic adult mice. Transgenic animals express ELC in both NTg and mutant forms and purified protein samples reflect this heterogeneity. Quantitative characteristics for the WT and NTg species are practically identical, hence we do not treat them as separate species. WT versus A57G or WT versus E143K sometimes show stark contrasts (measured from the heterogeneous samples), so we will sometimes want to distinguish their relative contributions.

Earlier work with N-terminus-truncated ELC indicated the heterodimer of native and truncated ELC to be a distinct species that behaved similarly to the modified homodimer [9]. Overlapping motility characteristics of the modified homodimer with the heterodimeric myosins in E143K and A57G suggest the modified species characteristics will dominate all motility measurements. The Qdot assay in this manuscript is used to characterize the effect of load on the homogeneous species. Contrasting responses of heterogeneous A57G and E143K samples to load are qualitatively apparent and sufficient for our descriptive purposes. The ensemble average step-sizes and duty-ratios derived from the Qdot data are used in a calculation of motility velocity (see below), but differences for these quantities between the heterogeneous A57G and E143K samples have negligible influence on motility velocity outcomes.

Motility velocity, *s*_m_, for a binary mixture of cycling myosins is nonlinear in their relative fraction in bulk and determined mainly by action of the slowest-moving motor [29]. Motility velocity in the presence of load can be expected to behave similarly. We generalize the previous work to include load permitting us to estimate elasticity. We find that the nonlinearity in velocity introduced by the mixed species is negligible because motility velocities between minor and major species are small, but the estimate for elasticity is a unique and valuable characterization that justifies the extra work. The full calculation justifies our suggestions above that the heterogeneous sample reflects the material properties of the major species.

We assume myosin heads in a dimer function independently. This assumption applies to parameters determined for actin-activated ATPase and *in vitro* motility. We do not need this assumption for the Qdot assay parameters for the reasons discussed above. Below we address categorically how the mixed species affects ATPase and *in vitro* motility.

### 2.12. Actin-activated ATPase

Myosin monomers function independently in the actin-activated ATPase assay. ATPase characteristic quantities, *Q* (*V*_max_ or *K*_m_), estimated for a mixture of homogeneous mutant, Mu, and NTg species are2.15
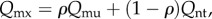
for subscripts mx, mu and nt indicating the mixed, mutant and NTg species, respectively, and *ρ* = [Mu]/([Mu] + [NTg]) the mole fraction of mutant in the mixture with NTg. The NTg and WT species have identical actin-activated ATPases to within error [9] implying we can replace NTg with the WT species-derived parameters.

### 2.13. *In vitro* motility

Motility velocities for the NTg and WT species are identical to within error [9], implying we can replace NTg with the WT species motility velocity. Cuda *et al.* [29] derived an expression for myosin-based motility velocity for a mixture of two actively cycling myosins with rapid (r) and slow (s) motility velocities. The two-state model, involving an actin-detached state and actin-attached force producing state, is similar to that proposed by Huxley [2]. From eq. A.2 in [29] and slightly generalized to include a frictional force (load) on actin proportional to velocity, unique step-size for each homogeneous species and unique duty ratio for each species has2.16
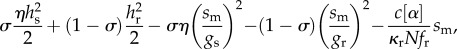
for s_m_ the myosin motility velocity for the mixture of slow and rapid myosins, *σ* = [M_s_]/([M_r_] + [M_s_]) the slow myosin fractional concentration, *η* = *κ*_s_*f*_s_/*κ*_r_*f_r_* for *κ*_r(s)_ the myosin elasticity and *f*_r(s)_ the ensemble average duty ratio for rapid (slow) myosin, *g*_r(s)_ the rapid (slow) myosin actin detachment rate, *h*_r(s)_ the rapid (slow) ensemble average myosin step-size, l the distance between actin-binding sites, c([*α*]) from equation (2.3), and *N* the total number of myosin motors moving actin. We combine constants related to friction such that2.17
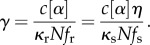


We assume that the dimeric myosin heads are functioning independently when moving actin and that torque from the motor domain is constant while elasticity and step-size vary suggesting *η* = (*h_r_/h_s_*)(*f*_s_/*f_r_*) for *h* computed from average step-size values. Solving equation (2.16) for *s*_m_,2.18
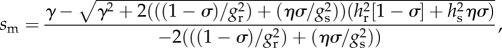
Unloaded actin has *γ* = 0, while all quantities in equation (2.18) are known except for detachment rates. We used this condition to estimate *g* for all the myosin species.

We estimate the strain-dependent rates using the two-state model where2.19
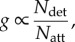
for *N*_det_ the number of detached myosin motors, *N*_att_ the number of attached myosin motors with *N* = *N*_det_ + *N*_att_. Comparing detachment rates for strained myosin due to the application of external frictional force *F* and unstrained myosin2.20
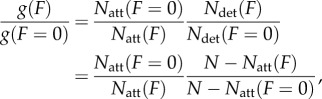


Ensemble averaged duty ratios for the cardiac myosins indicate that *N*_att_ is always small compared with *N* such that2.21
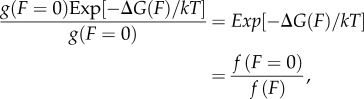
for Δ*G*(*F*) = (1/2) *F* Δ*x* and Δ*x* the myosin strain due to *F*. We use Δ*x* as a free parameter to satisfy equation (2.21) for each myosin species obtaining the expression for g(F). Given g(F), ensemble average step-sizes, ensemble average duty ratios and the measured loaded actin velocity, we can surmise the load-dependent *γ* from equation (2.16), thus indicating the load-dependent elasticity for each myosin species using equation (2.17). We plot the elasticity-proportional quantity *κ*N (ensemble elasticity) in units of pN nm^−1^. Total number of myosin motors, *N*, is approximately comparable among all species tested because myosin density in the motility assay is approximately equal for identical myosin bulk concentration.

## 3. Results

### 3.1. Expression levels of human ELC

**Figure 1.**
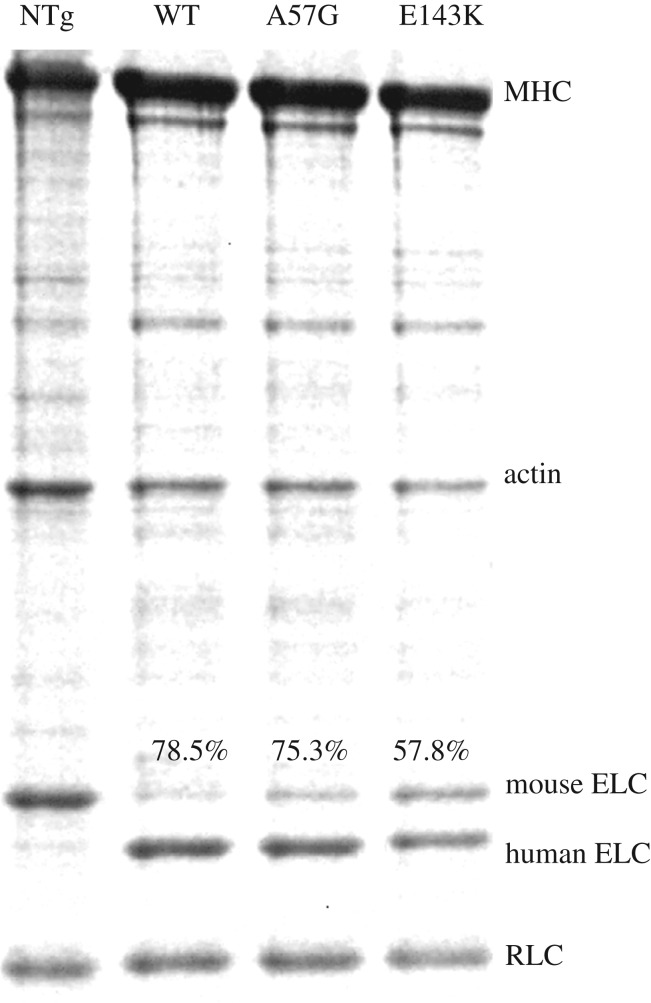
SDS-PAGE of nontransgenic (NTg) and transgenic WT, A57G and E143K myosin preparations. MHC is the mouse cardiac α-myosin heavy chain. Human versus total ELC is indicated with %.

Figure 1 shows the SYPRO Ruby stained SDS-PAGE gels for mouse *α*mys species. NTg is the nontransgenic mouse myosin. Staining intensities for native RLC and ELC compared with the human ELC species WT, A57G and E143K indicate 79% human native ELC, 75% human A57G and 58% human E143K, with the remainder the native mouse isoform.

Intact muscle data from the transgenic mouse models reflect the impact of the mixed ELC species; hence, we assess and report quantitative *in vitro* myosin characteristics from measurements on the mixed species. We show subsequently that *in vitro* results extrapolated to homogeneous A57G and E143K species leave unchanged qualitative conclusions based on the original data from mixed species.

### 3.2. Actin-activated ATPase of cardiac myosin

**Figure 2.**
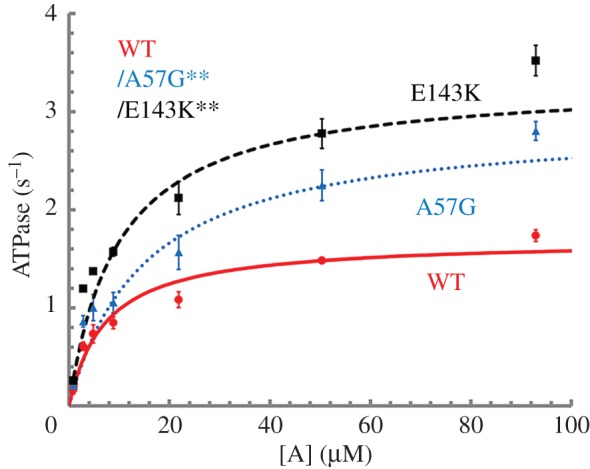
Actin-activated myosin ATPase versus actin concentration [A] for wild-type (WT) and mutants E143K and A57G. Error bars show standard deviation for sampling statistics given in table 1 and under experimental conditions given in Material and methods. Fitted curves use equation (1.1). Significance of ATPase rate versus [A] data in pairwise comparison for the WT and mutant species is indicated. They differ significantly with confidence level *p* < 0.01 as indicated by **.

Figure 2 shows the actin-activated ATPase versus actin concentration, [A], for WT (red circles), A57G (blue triangles) and E143K (black squares) with error bars indicating standard deviation. Actin-activated myosin ATPase on WT, A57G and E143K distinguished each protein species mainly by *V*_max_ with A57G and E143K nearly 2× higher than WT. Table 1 summarizes the Michaelis–Menten parameters and the sampling statistics for the data in figure 2.

**Table 1.** Average mechanical characteristics of transgenic myosins. Average values and standard deviation indicated for (*n*) replicates. ANOVA significance under the *p*< heading indicates confidence level for distinguishing WT/A57G and WT/E143K pairs. NA indicates not applicable for error derived from fitting equation (2.5) and using equation (2.6). ATPase characteristics for homogeneous E143K and A57G species were computed from the quantities listed in the table using equation (2.15). *V*_max_ for the homogeneous species of A57G or E143K is expected to be 3.30 or 4.51 1/s, while WT is unchanged. *K*_m_ for the homogeneous species of A57G or E143K is expected to be 11.7 or 17.6 µM, while WT is unchanged. In each case, the correction exaggerates the prevailing tendency in their difference from WT. The uncertainty scales similarly. Other characteristics for the homogeneous species are indicated in electronic supplementary material, figure S2. *V*_max_ and *K*_m_ are Michaelis–Menten constants defined in equation (2.1).

	WT	A57G	E143K	*p*<
actin-activated myosin ATPase				
*V*_max_ (1/s)	1.69 ± 0.06 (*n* = 6)	2.90 ± 0.16 (4)	3.32 ± 0.01 (2)	0.01
*K*_m_ (μM)	7.22 ± 0.54	15.0 ± 3.21	9.81 ± 0.49	—
peak *in vitro* motility velocity for unloaded actin				
*s*_m,max_ (μm s^−1^)	0.81 ± 0.05 (*n* = 32)	0.91 ± 0.05 (38)	0.74 ± 0.05 (15)	0.01
isometric force in pN				
*F*_iso_ (equation (2.2))	514.7 ± 34.7 (*n* = 11)	699.0 ± 52.4 (7)	373.0 ± 37.4 (7)	0.01
*F*_iso_ (equation (2.5))	413.2 ± 4.9	488.3 ± 7.5	325.8 ± 6.6	0.01
peak power at load				
*P*_max_ (fW)	0.049 ± 0.005	0.060 ± 0.007	0.032 ± 0.006	n.a.
at load (pN)	141.0 ± 6.9	155.8 ± 8.5	114.0 ± 10.1	n.a.

We tested the significance of data in figure 2 using two-way ANOVA with factor 1 proteins WT, A57G and E143K, and factor 2 the actin concentration [A]. We find the datasets differ significantly with confidence levels *p* < 0.01 (**) in pairwise comparisons of WT with mutants. The HCM implicated ELC mutants substantially impact myosin kinetics by speeding up actin-activated hydrolysis without distinguishing weak actin binding.

### 3.3. *In vitro* motility

Electronic supplementary material, figure S1 shows motility velocity, *s*_m_, versus myosin bulk concentration, [M], for WT (red circles), A57G (blue triangles) and E143K (black squares) with error bars indicating standard deviation. Motility velocity increases with increasing *α*mys bulk concentration until reaching maximum at 0.14–0.20 µM and then slightly decreasing. Peak motility velocities and sampling statistics are given in table 1. The motility velocities versus [M] are similar for each species and compare favourably with earlier measurements of unloaded velocities for the WT species [9]. The ELC mutations do not significantly impact the movement of unloaded actin.

### 3.4. Loaded Qdot assay models the working muscle

**Figure 3.**
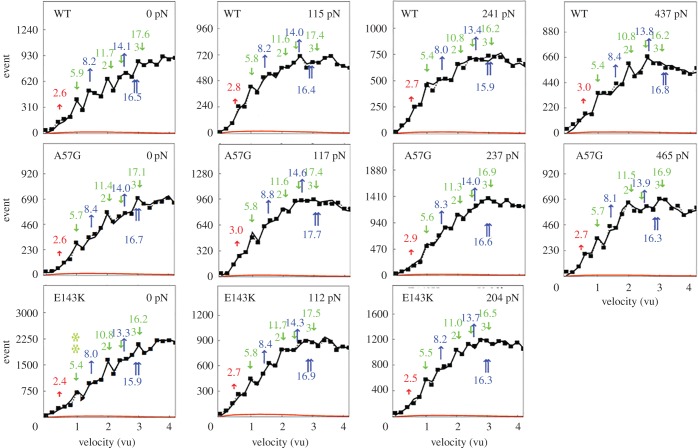
Event versus velocity histograms (solid squares) for WT, A57G and E143K myosins and for frictional actin loading, *F*_f_, from 0 to 465 pN. The solid red line is the baseline due to thermal/mechanical fluctuations that was subtracted from the raw data to give the data points at solid squares. Black lines are simulations conducted as described in ‘Material and methods’ and used to estimate step-size (at arrows) and step-frequency (figure 4). Natural velocity units (vu) have 1 vu = (*d*_I_/Δ*t*) for *d*_I_ the intermediate step-size (green down arrow at approx. 5–6 nm) between the short (red up arrow at approx. 2–3 nm) and long (blue up arrow at approx. 8–9 nm) step-sizes. Step-sizes have standard deviation of approximately 0.5 nm for replicates described in ‘Statistics’. The intermediate step-size for the E143K species under 0 load differs significantly from equivalent WT species with confidence level *p* < 0.01 as indicated by **. Other step-sizes are not significantly different for confidence level *p* < 0.05.

The Qdot assay has labelled actin filaments approximately 1 µm long translating over surface-bound mouse *α*mys. Figure 3 shows the Qdot assay event–velocity histogram pooled data from 16 to 55 acquisitions for the WT, A57G and E143K species, and for increasing load as indicated in pN. Columns are nearly equal frictional loads for the rows with identical transgenic myosins. The event–velocity histograms cover the low velocity domain of 0–4 natural velocity units (vu) where (*d*_*I*_/Δ*t*) = 1 for d_I_ the intermediate step-size of ∼5–6 nm and frame capture interval Δ*t* = 45 ms. Summary data (solid squares) have the baseline contributed by thermal/mechanical fluctuations (red solid line) already subtracted to give motility due to myosin activity. Peaks or inflection points appearing below 2 vu are short (short red up arrow or S), intermediate (*d*_I_ and longer green down arrow or I) and long (longest blue up arrow or L) step-sizes in nm. Green and blue arrows also indicate a few of the unitary step combinations. Step-sizes are indicated by the colour-coded numbers nearest the arrows. Step-size significance was tested using one-way ANOVA comparing WT, A57G and E143K species. The intermediate step-size for the E143K species under 0 load differs from the equivalent WT species with confidence level *p* < 0.01 as indicated by ** in figure 3. The significance suggests a shorter intermediate and average step-size for E143K compared to WT.

**Figure 4.**
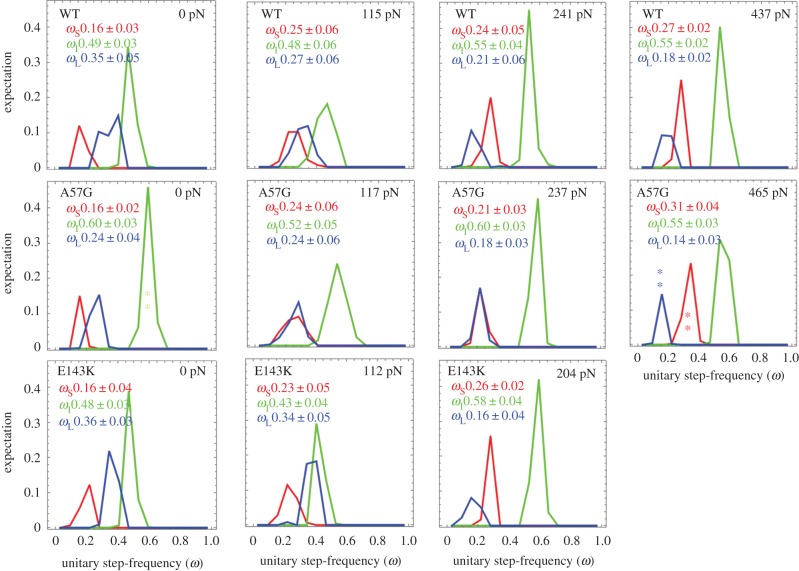
The step-frequencies for the WT (top row), A57G (middle row) and E143K (bottom row) under frictional loads indicated in each panel in pN units. Curves are derived from simulation of the corresponding event–velocity histograms in figure 3 and as described in the Qdot assay event–velocity histogram simulation’ section. Columns are equivalently loaded actin for each transgenic protein. Errors are SDs for replicates described in ‘Statistics’. Intermediate step-frequency (green) for the A57G species differs significantly from equivalent WT species with confidence level *p* < 0.01 for no load and as indicated by **. Short (red) and long (blue) step-frequencies for the A57G and WT species also differ significantly with confidence level *p* < 0.01 under maximum load and as indicated by **.

Figure 4 shows the step-frequency (*ω*) expectations, expectation values and SDs for the S, I and L unitary steps derived by simulation from each event–velocity histogram in a one-to-one relationship with the panels in figure 3. The expectation curves indicate the relative probability for step-frequency along the abscissa. The area under the colour-coded curves for the S, I and L steps equals expectation values *ω*_S,_ *ω*_I_ and *ω*_L_, respectively. The sum *ω*_S__+_ *ω*_I__+_ *ω*_L_ = 1 in each panel. Step-frequencies in figure 4 for F_f_ = 0 indicate WT and E143K are statistically identical, while A57G favours the 5 nm step with a step-frequency of approximately 60% compared with approximately 50% in WT and E143K. The difference is significant at confidence level *p* < 0.01 (indicated by **). The canonical step-frequencies of 13, 50 and 37% observed in WT and E143K shift in favour of the 5 nm step in A57G. We attribute this to the perturbation of the actin-binding ELC N-terminus by the mutation at A57. Earlier work demonstrated that the step-frequencies for the 5 and 8 nm steps were similarly modulated by truncation of the ELC N-terminus [9]. Direction and amplitude of the step-frequency adjustment for the truncated ELC is identical to within error to that shown in figure 4 for A57G.

Step-size modulation for the E143K mutant (figure 3) and step-frequency modulation for the A57G mutant (figure 4) are mechanical manifestations of the mutations detected in unloaded conditions (first column in each figure). They cause average step-size to shorten either directly by lowering the intermediate step-size in E143K or by the failure of the ELC N-terminus to bind actin in a timely manner and so frequently failing to complete the 8 nm step-size in A57G. Previous work on E143K with the Qdot assay in unloaded conditions indicated its step-size was not significantly different from WT step-size [18]. The analysis of the older data together with additional Qdot assay data from both E143K and WT species implies the new assessment stated here.

Step-frequencies in figure 4 indicate the 5 nm step-size is always predominant; however, at the highest loads the second most frequent step-size reverses from 8 to 3 nm repeating a down-shifting manoeuver first observed *in vivo* from cardiac myosin in zebrafish embryo hearts [12]. Step-frequencies for the 3 and 8 nm step-sizes are significantly different in WT, A57G and E143K at a confidence level of *p* < 0.01. Cardiac myosin replaces 5 with the 3 nm step as the predominant step at isometric contraction in the zebrafish heart. This does not happen *in vitro* possibly because viscoelastic drag on actin movement imposed by α-actinin does not impose the isometric condition [24].

**Figure 5.**
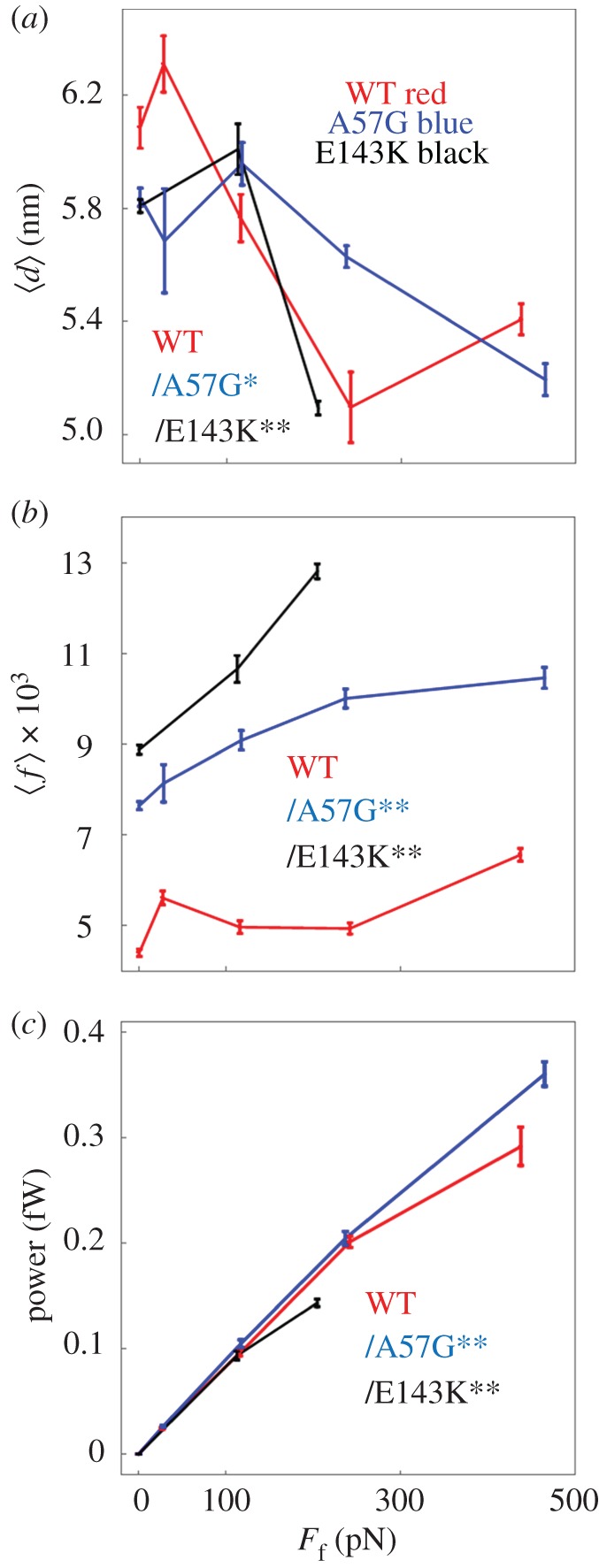
*In vitro* single myosin (*a*) average step-size 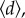 (*b*) average duty ratio and (*c*) power for WT (red), A57G (blue) and E143K (black) *α*cardiac myosin measured by the Qdot assay under a retarding force. Data points are derived from simulation of the corresponding event–velocity histograms in figure 3 and as described in ‘Qdot assay event–velocity histogram simulation’. Errors are standard error of the mean for the 16–55 replicates described in ‘Statistics’. Data in pairwise comparison for the WT and mutant species are indicated for each panel. They differ significantly with confidence level *p* < 0.05 or 0.01 indicated by * or **.

The WT and mutant myosins differ on how they accomplish the 8 to 3 nm step-size probability reversal with A57G requiring a higher loading force than WT or E143K. Figure 5*a* shows the average step-size, 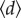, versus load. All species down-shift with loading; however, A57G consistently lags WT and E143K suggesting the mutation diminishes load-sensitivity. Figure 5*b* shows duty-ratio versus load. Control WT has a lower and flat duty ratio with increasing load implying that the fraction of force producing myosins during contraction is low and static. Compared with WT, A57G and E143K have high and rising duty ratio with increasing load implying that the fraction of force producing myosins is higher and growing during contraction phase. Figure 5*c* shows larger power production for A57G indicating hypercontractility. Myosin hypercontractility associates with HCM disease [30] but for reasons also including secondary effects induced by heart tissue remodelling [16]. For the same reasoning, hypocontractility in E143K (again panel c) is unexpected and suggesting E143K unitary force is compromised given that it has higher duty ratio but produces less work. One way for this to happen is if E143K reduces lever arm rigidity. A57G and E143K positions in ELC fit the scenario where A57G directly impacts ELC N-terminus actin binding and load-sensitivity during the active cycle, while E143K passively reduces lever arm rigidity. We tested significance of data in figure 5 using two-way ANOVA with factor 1 proteins WT, A57G and E143K, and factor 2 the frictional force *F*_f_. We find that these data differ significantly for each pairwise comparison of WT with mutants and with confidence levels *p* < 0.01 (**) or *p* < 0.05 (*).

Step-frequency modulation is the signature characteristic of the ELC N-terminus actin-binding interaction [9]. Recent work on the *in vivo* zebrafish heart suggests that the ELC N-terminus possesses strain-sensing characteristics when actin bound that has a ratchet-like selective resistance to movement in the direction of the loading force, while permissive to movement in the direction of the contractile force. Consequently, the step-frequency modulation caused by the loading of the actin filament in the myosins tested here (figure 4) implies a role for the ELC N-terminus actin binding in the response of single myosins to load. Here, it causes 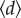 to shorten as load increases by up-modulating shorter 3 and 5 nm step-sizes at the expense of the longer 8 nm step-size. We introduced a four-pathway model for the cardiac myosin contraction cycle that rationalizes the effect of load on 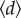 in live zebrafish [12]. In the next section, we apply the same model to Qdot assay data illustrating the close correlation between *in vitro* and *in vivo* contexts of myosin-based contraction.

### 3.5. Contraction cycle four-pathway model

**Figure 6.**
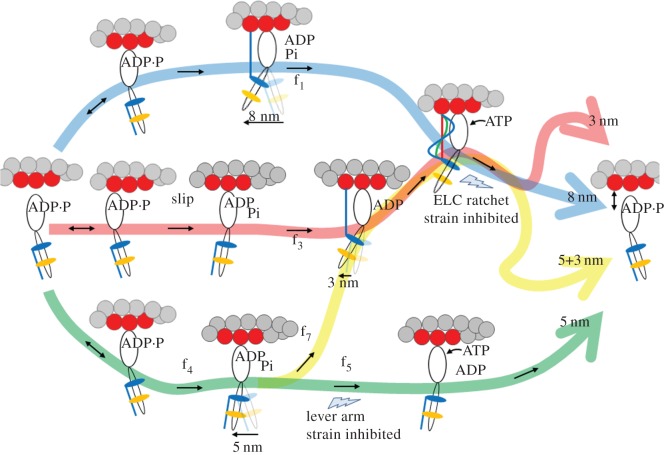
The four-pathway network for *in vivo* and *in vitro* cardiac myosin unitary steps. Myosin powerstroke has two sequential steps with Pi release driving the larger lever arm swing (nominal 5 nm step-size) followed by the ELC N-term binding actin and ADP release driving the smaller lever-arm swing (3 nm step-size). In the blue pathway with the 8 nm step-size, large and small steps are tightly coupled for the maximum lever-arm swing. In the bifurcated green/yellow pathway, Pi release with the 5 nm step is not immediately followed by ADP release and the 3 nm step due to the lever arm strain inhibition at the lower thunderbolt. The delay allows the powerstroke a choice to terminate with myosin detachment for a terminal 5 nm step-size (terminal half of green path) or complete the second 3 nm step (5 + 3 nm step-size via the yellow path). In vivo data provided evidence for a solo-3 nm step-size (red pathway) [12]. We propose in the figure that the myosin in this pathway slips to releases Pi without net forward movement, but then the ELC N-term binds actin permitting ADP release and completion of a solo-3 nm step. Myosin flux values through each pathway are f_1_ (blue pathway), f_3_ (red), f_7_ (yellow), f_4_ (green main channel) and f_5_ (green terminus) using nomenclature from [12]. Relative flux values are listed in table 2. The ELC-ratchet strain activated filter at upper thunderbolt regulates the solo-3, 8 and 5 + 3 nm pathways (red, blue and yellow, respectively). ELC ratchet strain inhibits ATP binding and ELC N-terminal detachment from actin maintaining tension at peak isometric force. The upper and lower lightning bolts indicate strain-regulated checkpoints that modulate step-frequencies for quickly responding to changing force-velocity demands.

Earlier work, using the unloaded *in vitro* Qdot assay on *β*mys [8] combined with the *in vivo* single cardiac myosin imaging of zebrafish embryo hearts [12], suggested a four-pathway network summarized in figure 6 for generating 8 (blue pathway), 5 (green), 5 + 3 (yellow) and solo-3 (red) nm myosin step-sizes. Earliest work using the unloaded Qdot assay alone identified the 8 (blue), 5 (green) and 5 + 3 (yellow) nm step-size pathways [8]. The *in vivo* zebrafish system added the context of the intact auxotonic and near-isometric muscle that identified the solo-3 nm step-size (red) pathway [12].

**Table 2.** Myosin flux through the four-pathway network. Flux through the blue pathway in figure 6 corresponds to the 8 nm step (*f*_1_), green pathway to the 5 nm step (*f*_5_), red to the solo-3 nm step (*f*_3_) and yellow to the 5 + 3 nm step (*f*_7_). Optimized flux values uniquely satisfy equality constraints from flux conservation (e.g. *f*_4_ = *f*_5_ + *f*_7_ and *f*_4_ = *x*_I_, see equation (2.14)), and inequality constraints (e.g. *f*_3_ ≤ *x*_S_, see equation (2.14)). Flux constraints and flux estimation are discussed for the full model in figure 8 and table 1 from Burghardt *et al*. [12]. Average flux values are indicated with standard deviation for the 16–55 replicate Qdot assays (see ‘Statistics’). Significance values in the *p*< rows are for the WT, A57G and E143K species are indicating the one-way ANOVA comparison of flux for 0 and maximum load cases. *p* < 0.01 implies the step-size column has flux values that differ due to load, while — indicates no significant difference. Abbreviations: *F*_r_ is the frictional loading force (equation (2.4)).

*F*_f_ (pN)	*ω*_0_	*f*_1_ (8 nm)	*f*_5_ (5 nm)	*f*_3_ (solo-3 nm)	*f*_7_ (5 + 3 nm)
WT					
0	0	39 ± 4	43 ± 4	6 ± 3	12 ± 3
115	0.23 ± 0.06	30 ± 6	42 ± 7	15 ± 5	13 ± 4
241	0.23 ± 0.05	23 ± 6	50 ± 6	14 ± 5	13 ± 4
437	0.43 ± 0.08	20 ± 2	51 ± 3	20 ± 4	9 ± 5
*p*<		0.01	0.01	0.01	—
A57G					
0	0	27 ± 4	56 ± 3	6 ± 1	11 ± 1
117	0.25 ± 0.09	27 ± 6	45 ± 7	15 ± 5	13 ± 4
237	0.32 ± 0.08	20 ± 3	56 ± 3	12 ± 4	12 ± 3
465	0.41 ± 0.07	15 ± 3	51 ± 4	24 ± 5	10 ± 5
*p*<		0.01	0.01	0.01	—
E143K					
0	0	40 ± 2	42 ± 4	7 ± 2	11 ± 3
112	0.13 ± 0.11	37 ± 4	37 ± 5	16 ± 5	10 ± 4
204	0.27 ± 0.06	19 ± 4	52 ± 3	15 ± 3	14 ± 4
*p*<		0.01	0.01	0.01	—

In figure 6, weak actin-binding myosin reversibility (indicated with ↔) preemptively avoids pathways that do not complete a cycle under increasing load. Under loaded conditions, flux through the cycle is checked at two strain inhibited points. The traditional lever arm strain checkpoint inhibits ATP dissociation of actomyosin following the 5 nm step-size (green path) sending flux towards the 3 nm step-size (yellow branch). The new ELC-ratchet strain activated filter regulates the solo-3, 8 and 5 + 3 nm pathways (red, blue and yellow). The latter senses tension in the actin bound ELC N-terminus such that the slack ELC extension (blue, when muscle is unloaded or rapidly shortening under low load) allows ATP binding and quick detachment from actin to complete the cycle, the moderately tense ELC extension (green, when muscle is under moderate loads approaching isometric) partially inhibits ATP binding and detaches slower from actin to exert static myosin tension contributing to force on slowly translating muscle filaments, and the maximally tense ELC extension (red, when muscle is near isometric) inhibits ATP binding and detaches slowest from actin to exert static myosin tension contributing more of the force on the static muscle filaments. The ELC-ratchet filters out longer step-sizes as tension rises. Strongly bound myosins contributing static tension are the force-bearing 0-length step-size myosins not explicitly depicted in figure 6 (see rather fig. 8 in [12]). Modulating flux through two strain-dependent steps with different inhibitions adjusts average step-size. The favoured pathway at high tension (greater than 80% of cycles) involves the solo-3 nm step via the red pathway. It releases Pi without net forward movement probably by slipping at high tension but then completes a 3 nm step. Slip distances of 2–8 nm were observed in synthetic myofilaments under tension (see fig. 1 panels in [31]; larger slips were observed earlier [32]). This pathway is populated even at the highest tension implying the ELC ratchet strain-based checkpoint is less inhibiting than the lever arm strain-inhibited checkpoint in near-isometric contraction [33].

**Figure 7.**
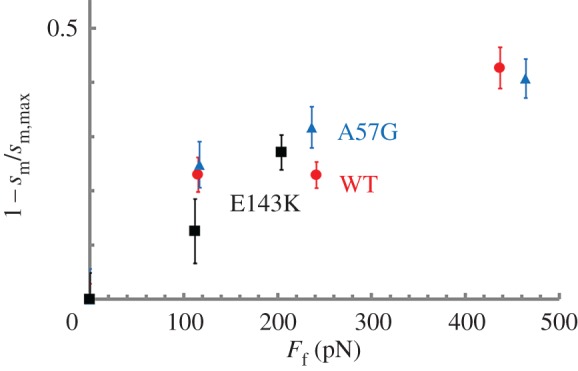
The actin filament fraction under isometric loading versus loading force estimated from the motility of Qdot labelled actin and equation (2.13). Errors are standard deviation for the 16–55 replicates described in ‘Statistics’.

Computing myosin flux through the 4-pathway network in figure 6 uses step-frequency expectations {*ω*_S_, *ω_Ι_*, *ω*_L_} from figure 4 and the step-frequency expectation for the 0-length step-size myosins, *ω*_0_, substituted into equation (2.14) to form step-frequency fractions, *x*_0_, *x*_S_, *x*_I_ and *x*_L_ as described in [12]. Figure 7 shows *ω*_0_ for the WT, A57G and E143K species plotted over the frictional drag force imposed by α-actinin. Table 2 summarizes *ω*_0_ (from figure 7) and the computed flux values. Table 2 caption briefly describes how the myosin flux through the pathways is computed. Flux values confirm that all myosin species autonomously down-shift ensemble displacement with increasing resistive load by remixing their three divergent unitary step-sizes with changed frequencies (flux) in a mechanism paralleling that observed *in vivo*.

### 3.6. Impact of mutation on ensemble myosin force/velocity

**Figure 8.**
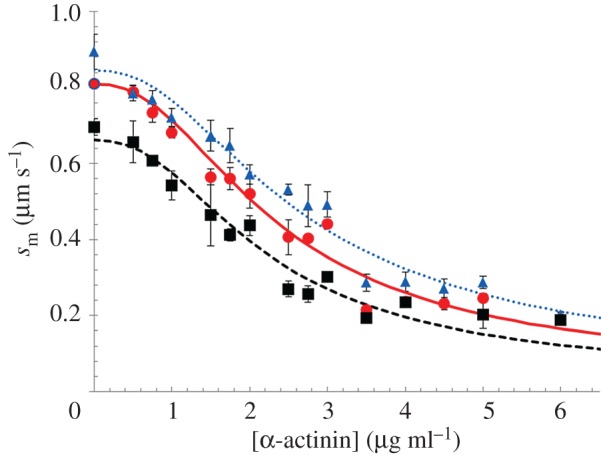
Motility velocity, *s*_m_, for myosin isoforms WT (red circles), A57G (blue triangles) and E143K (black squares) versus α-actinin concentration. Sample sizes for the SDs indicated by error bars are WT (*n* = 11), A57G (*n* = 7) and E143K (*n* = 7) for *n* the number of acquisitions corresponding to 1 *in vitro* motility movie per acquisition. Fitted curves are based on equation (2.2) with the friction coefficient c([*α*]) converted to calibrated units (pN s µm^−1^) using equation (2.3) and ensemble myosin driving force *F*_d_ equal to isometric force.

Figure 8 shows motility velocity, *s*_m_, versus α-actinin concentration for WT (red circles), A57G (blue triangles) and E143K (black squares). Fitted curves are based on equation (2.2) with the friction coefficient c([*α*]) converted to calibrated units (pN s µm^−1^) using equation (2.3) and ensemble myosin driving force *F*_d_ equal to isometric force. Estimated isometric forces deduced from the data in figure 8 and using equations (2.2) and (2.3) are indicated in table 1 for each myosin species. Error bars in figure 8 indicate standard deviation for sampling statistics given in the figure caption.

**Figure 9.**
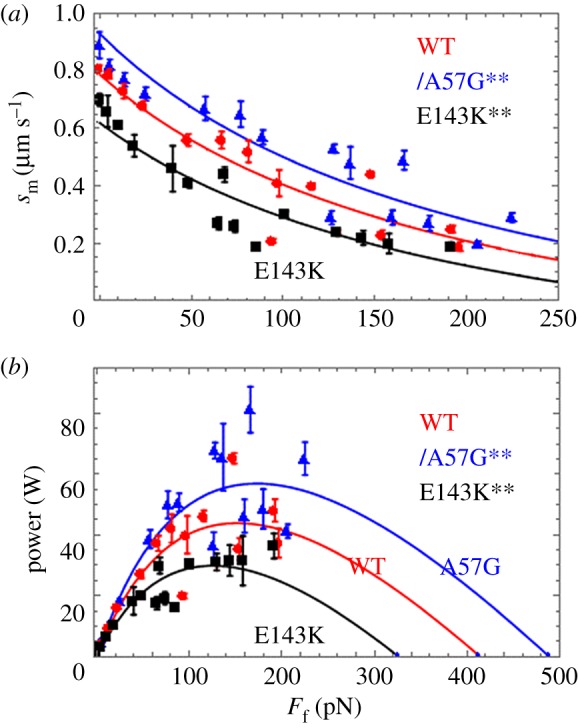
(*a*) Motility velocity (*s*_m_) and (*b*) power in aW for myosin isoforms WT (red circles), A57G (blue triangles) and E143K (black squares) versus calibrated frictional force, *F*_f_, exerted by the α-actinin. Sample sizes for the SDs indicated by error bars are WT (*n* = 11), A57G (*n* = 7) and E143K (*n* = 7) for n the number of acquisitions corresponding to 1 *in vitro* motility movie per acquisition. Significance testing of *s*_m_ versus *F*_f_ data in (*a*) and power versus *F*_f_ data in (*b*) in pairwise comparison for the WT and mutant species indicates that they differ significantly with confidence level *p* < 0.01 denoted by **. Fitted curves are based on equations (2.5) and (2.6) (Hill equation) for (*a,b*).

Figure 9*a* shows *s*_m_ and 9*b* shows power (*s*_m_ × *F*_f_) versus loading frictional force, *F*_f_ exerted by α-actinin in piconewtons (pN) from equation (2.4), for WT (red circles), A57G (blue triangles) and E143K (black squares). Power units are attowatts (aW). Fitted curves are based on equations (2.5) and (2.6) (Hill equation). The isometric forces obtained from fitting the data in figure 9 and indicated by the *x*-intercept in figure 9*b* are also summarized in table 1 for each myosin species. Error bars in figure 9 indicate standard deviation for sampling statistics given in the caption. We tested significance of data in figure 9*a,b* using two-way ANOVA with factor 1 proteins WT, A57G and E143K, and factor 2 the frictional force *F*_f_. We find the curves differ significantly with confidence levels *p* < 0.01 (**) for each pairwise comparison of WT with mutant. The HCM implicated ELC mutants substantially and differently change the *in vitro* force–velocity characteristics of the cardiac myosin with A57G enhancing and E143K diminishing power generated compared with WT.

### 3.7. Impact of mutation on ensemble myosin elasticity

We estimated myosin ensemble elasticity for each species as described in equations (2.16)–(2.21). Myosin elasticity is a fundamental structure/function characteristic of the motor that depends principally on the loaded *in vitro* data from the last section and uses the unitary myosin step-size and duty-ratio quantities measured with the Qdot assay.

The mixture including E143K and NTg corresponding to the slow (s) and rapid (r) myosins has slow myosin fraction, *σ* = 0.578 (figure 1), average step-sizes *h*_s_(E143K) = 5.8 nm and *h*_r_(NTg) = 6.1 nm (average step-size from figure 5*a*), giving for the homogeneous species the actin-detachment rates in the absence of external forces, *g*_r_(NTg) = 0.188 1/s and *g*_s_(E143K) = 0.156 1/s. The mixture including A57G and NTg has A57G corresponding to the rapid myosin with *σ* = 1–0.753 (figure 1) and *h*_r_(A57G) = 5.9 nm (figure 5*a*), giving for the homogeneous species *g*_r_(A57G) = 0.225 1/s. Average step-size and consequently *η* are load dependent from figure 5*a*. Likewise, detachment rates decrease with load due to strain inhibition as implied by the scheme in figure 6 and estimated using equation (2.21).

Actin velocities measured from mixed species at various constant loads are the left-hand side of equation (2.18) that we solve for *γ*. Estimates for *γ* are likewise load dependent and indicate an absolute load-dependent elasticity for each myosin species using equation (2.17). We use this load-dependent *γ* to compute velocity for the homogeneous species when *σ* = 1 for E143K or *σ* = 0 for A57G. Homogeneous species force is then computed from the homogeneous species velocity using the friction coefficient from equation (2.3). The favourably high transgenic ELC expression in the mouse hearts causes slight or inconsequential differences between the homogeneous and mixed species with the homogeneous A57G velocities and power slightly higher than the corresponding mixed species, while homogeneous E143K velocities and power slightly lower at low *F*_f_ and slightly higher at high *F*_f_ compared with the corresponding mixed species. These data are summarized in electronic supplementary material, figure S2. Quantitative differences between the homogeneous and mixed data are indicated with the fitted curves using equations (2.5) and (2.6) and isometric forces indicated by the *x*-intercepts in electronic supplementary material, figure S2*b*.

**Figure 10.**
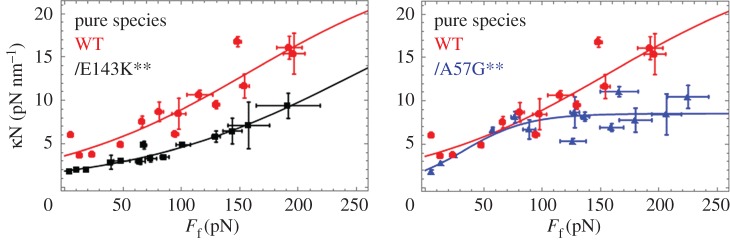
Ensemble elasticity *κ*N versus frictional loading force *F*_f_ for each homogeneous species computed using equations (2.3) and (2.17). Two-dimensional error bars indicate standard deviation for *n* = 5–51 trials at each data point. Pairwise comparison for the WT and mutant species show that they differ significantly with confidence level *p* < 0.01 indicated by **.

Figure 10 shows ensemble elasticity *κ* N versus *F*_f_ for each homogeneous species and computed as described in ‘Material and methods’. Fitted curves in figure 10 are formed from a logistic function given by3.1
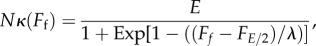
for *N* the total myosin motors binding the actin filament, *E* the peak ensemble elasticity, *F*_E/2_ the loading force where ensemble elasticity is *E*/2, and *λ* characterizing the steepest ascent at the midpoint *F*_f_ = *F_E_*_/2_ where maximum slope is *E*/4*λ*. WT elasticity is highest requiring the most force for given strain with *E* ≈ 25 pN nm^−1^ and *E*/4*λ* ≈ 0.08–0.10 (nm)^−1^. E143K is similar to WT species except that *F_E_*_/2_ shifts to higher force by approximately 100 pN. A57G is distinguished by *E* ≈ 9 pN nm^−1^, *E*/4*λ* ≈ 0.03–0.04 (nm)^−1^ and *F_E_*_/2_ shifted to lower force by approximately 100 pN compared to WT. We tested significance of data in figure 10 using two-way ANOVA with factor 1 proteins WT, A57G and E143K, and factor 2 the frictional force *F*_f_. We find the curves differ significantly for each pairwise comparison of WT with mutants and with confidence levels *p* < 0.01 (**). Overall, it suggests that A57G detaches from actin at lower force probably also causing lower maximum slope and the down-force shift in the elasticity curve. E143K up-force shift of *F*_E/2_ might reflect the loss in lever arm bending stiffness also suggested by its hypocontractility. Figure 10 curves show that we detect nonlinear myosin elasticity with loading analogous to that in skeletal myosin filaments and with the peak elasticity for WT and E143K compared with that from single skeletal myosin, suggesting an average of *N* = 10 myosin motors actively producing force [34].

Table 2 shows *ω*_0_, the amount of force bearing 0-length step-size myosins relative to all force-bearing myosins, rises with load. It implies active myosins are tending to reside in strongly actin-attached states as the load rises. Similarly, ensemble elasticity rises with load reasonably suggesting that strongly actin bound myosins are stiffer.

## 4. Discussion

In vitro motility and Qdot assays have myosin immobilized on a planar glass substrate under prismless total internal reflection illumination [35]. The myosin impels rhodamine–phalloidin or sparse Qdot labelled actin whose movement is detected by fluorescence. The high-throughput Qdot assay has nanometre-scale actin displacement measured using super-resolution microscopy [36] and negligible compliance to faithfully characterize the isolated myosin motor mechanics [9]. Prior work with unloaded actin in the Qdot assay indicated cardiac myosin moves actin with three distinct unitary step-sizes and with characteristic step-frequencies [8]. Here, we introduce various constant loads on the translating actin filaments and measure their effect on myosin step-size, step-frequency, motility and elasticity. In vitro loading emulates the cardiac ventriculum where a heartbeat circumscribes changing modes from relaxation, to auxotonic shortening, to isometric contraction.

Zebrafish embryo heart *in vivo* single myosin mechanical data indicated cardiac myosin down-shifts average displacement by remixing the three unitary step-sizes with changed step-frequencies [12]. Down-shifting occurs on the fly from a high-displacement/low-force transducer for high-velocity auxotonic shortening into a low-displacement/high-force transducer maintaining tension in near-isometric contraction. We report here qualitatively equivalent myosin down-shifting *in vitro* using the loaded Qdot assay. Data in figure 4 show the step-frequencies for transgenic mouse *α*mys with human ELC (WT) in the top row with loading force from α-actinin increasing from left to right. Increasing load remixes contributions from the longest and shortest step-sizes to reduce the average myosin step-size, 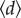, shown in figure 5. Likewise, average duty-ratio and power also in figure 5 indicate other systemic adaptations to the load resulting from the motor step-frequency adaptation indicated in figure 4. Overall, our data demonstrate that the recombination of step-size frequencies for specific loaded conditions adapts the myosin motor to a changing load demand in evident correspondence with the *in vivo* system. It implies that the varied combination of step-sizes for specific loaded conditions, first observed *in vivo*, does not require hierarchical or ensemble mechanical properties acquired by motor integration into whole muscle. It also implies, like the *in vivo* results [12], that the structural basis of the three step-sizes provided by the ELC ratchet is also involved in the myosin strain-sensitivity.

Nanometre displacement of actin by single myosin filaments containing multiple motors and under load indicated that single skeletal myosins have 2.5–8 nm unitary step-sizes by re-combination of 2.5 and 5.5 nm sub-steps corresponding to our 3 and 5 nm step-sizes [31]. It is proposed that the multiple motors on myofilaments manage coordinated force generation in skeletal muscle through their common attachment to the same actin filament via strain-dependent kinetics and that average step-size down shifts with load to approximately 3 nm. Implicit in this observation is that strain-dependent transition rates modify likelihood for different-length unitary step-sizes in the motor. This occurs in the absence and presence of external load and is caused by strain sensitivity altering step-size frequency (or remixing the three unitary step-sizes) implied by the model in figure 6. By the identical mechanism, we observed average myosin step-size up-shifting in individual actin filaments by raising unloaded motility velocity with more actomyosin attachments [37]. Uniquely, our model allows actin detachment after the 5 nm step that is unmistakable because of the unequal 3 and 5 nm step-frequencies [8] and the large fraction of the solo-3 nm step-size *in vivo* [12]. The latter is consistent but not required by the new *in vitro* data.

Significance of the ELC ratchet in familial heart disease is tested with two ELC mutants, A57G and E143K. They cause distinct disease phenotypes, occupy opposite ends of the myosin light chain sequence and differently affect single *α*mys mechanics. The A57G and E143K mutants cause hypertrophy in human hearts, while E143K is further differentiated as causing restrictive physiology. Mouse model hearts expressing the mutations have phenotypes likewise distinguishing their impact on function with A57G recapitulating human HCM-enhanced cardiac output and stroke work [16], while E143K recapitulates important aspects of human restrictive cardiomyopathy including reduced cardiac output and stroke work, abnormalities in diastolic function, and fibrosis [18]. Cardiac power output follows the stroke work with A57G [16] and E143K [18] because each mouse group has equal heartbeat rate. In vitro, myosins containing the A57G or E143K mutations have approximately 2× larger *V*_max_ than control WT but unaffected *K*_m_ in actin-activated myosin ATPase. Motility velocity is little affected (table 1). Unique differences between A57G and E143K emerge with the imposition of a load. In vitro single myosin and ensemble myosin motility data force/velocity measurements summarized in figures 5*c* and 9*b* indicate power up-regulation in A57G and down-regulation in E143K compared to WT, thus following the *in vivo* trends and consistent with myosin autonomy.

Duty ratio versus load for the A57G (figure 5) is displaced upward compared with WT by the constant and larger *V*_max_, while curve shapes differ from their contrasting dependence on step-frequency versus load. The effect of mutation on duty ratio versus load is similar but amplified in E143K due to the larger change in *V*_max_. A57G and E143K have high and rising duty ratio with increasing load compared with WT implying their ensemble will have more force producing myosins. If actively cycling versus super-relaxed [38] myosins re-equilibrate in the sarcomere during the phases of a heartbeat, the shift to strongly actin bound myosins implied by the duty ratio has the ELC mutants recruiting more super-relaxed myosins to the active state during the contraction phase than the native myosins. A role for disruption of the super-relaxed state in cardiac hypertrophy was suggested previously [30]. Either way, more ATP consumption impacts health.

In the unloaded Qdot assay, the A57G and E143K mutants stand out due to their altered step-frequencies and step-size compared with control WT, respectively. Step-frequencies for A57G resemble those observed from N-terminal-truncated ELC myosins in porcine or mouse cardiac myosin [9] and imply A57G disables the ELC ratchet. Figure 10 shows lower A57G peak ensemble elasticity compared to WT, implying a more compliant strongly actin bound myosin possibly from the reduction in the ELC N-terminal extension cross-links in the ensemble. The intermediate step-size of approximately 5 nm for E143K is significantly shortened compared to control. We also noted that 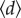 for the unloaded mutants are both less than that for WT (figure 5*a*). For A57G, it is due to step-frequency modulation by the mutation (figure 4 middle row). For E143K, it is due to step-size reduction (figure 3 bottom row). Overall positioning of substitutions A57G and E143K in ELC seems to fit a scenario with A57G in the N-terminus directly impacting actin binding during the active cycle and E143K passively reducing lever arm rigidity probably because of weaker E143K binding to myosin [39]. A57G also weakens ELC binding to myosin but to a lesser extent than E143K because its *K*_D_ for lever arm binding is closer to the native ELC [39]. Higher duty ratio overcompensates a potentially lower unitary force in A57G giving increased average power.

Imposition of load on the actin filament is reflected in the step-frequencies for each protein species in figure 4. WT, A57G and E143K all respond distinctively to the increasing load; however, at the highest loading force they similarly favour the 3 over the 8 nm step. The myosin flux through the four-pathway network in figure 6 gives the detailed accounting for the step-size adaptation to load that tallies an expanded set of step-size frequencies for each myosin species and loading condition. Table 2 summarizes flux through the system in 4 step-size categories that separate probability for the unique solo-3 nm step-size from the 3 nm step-size coupled to its antecedent 5 nm step. The tallies show significant incremental decreases in the 8 and increases in the solitary 3 nm step-frequencies, while the 5 + 3 nm step-frequencies are about the same. The 5 nm step-frequency uniquely distinguishes A57G from WT and E143K probably due to its anomalous ELC ratchet.

## 5. Conclusion

*In vitro* results from the loaded Qdot assay presented here and earlier *in vivo* results [12] indicate that varied step-size combinations for specific loading conditions do not require hierarchical or ensemble mechanical properties acquired by motor integration into whole muscle. We indicate evident and quantifiable complementarity between *in vivo* and *in vitro* systems by comparing single myosin mechanics in identical step-size and step-frequency representations of these systems. The *in vitro* assay reliably replicates core aspects of the whole heart, thus simplifying future efforts to broadly mechanically characterize inheritable cardiac diseases implicating the cardiac motor. WT, A57G and E143K variants down-shift their step-size under load just like their native *in vivo* counterpart in embryonic zebrafish. A57G differs from the other species based on how it accomplishes the 8–3 nm step-size frequency reversal by requiring a higher loading force. Step-size down-shifting occurs by affecting the selection of the different length unitary steps by way of two embedded regulatory mechanisms, one well known to inhibit ADP release due to lever arm strain and a second novel mechanism inhibiting actin detachment due to strain on the ELC N-terminus actin-binding extension. The A57G mutant affects the latter to perturb strain regulation. In the native myosin, these competing regulators shape step-size selection for autonomic regulation of force–velocity.

## Supplementary Material

Supplementary material consists of 2 figures

## Data accessibility

All data used in this study are provided in summary form in the text or electronic supplementary material. Standard commercially available software was used for analysis as noted in the text or electronic supplementary material. Representative raw *in vitro* motility and Qdot assay data were deposited at Zenodo (search: 1eab4085710ef65e41e797805480adbd). Metadata accompanies the raw data identifying samples and parameters for image capture.
