# Supplementary material for: Single cardiac ventricular myosins are autonomous motors
Source: Open Biol. 2018 Apr 18;8(4):170240. doi: 10.1098/rsob.170240 (PMC5936712; doi:10.1098/rsob.170240)
Supplement: Supplementary material consists of 2 figures [file rsob170240supp1.docx]

Supplementary Material

Single Cardiac Ventricular Myosins are Autonomous Motors

Yihua Wang^1^, Chen-Ching Yuan^2^, Katarzyna Kazmierczak^2^, Danuta Szczesna-Cordary^2^, and Thomas P. Burghardt^1,3^

^1^ Department of Biochemistry and Molecular Biology, Mayo Clinic Rochester, Rochester, MN 55905

^2^ Molecular and Cellular Pharmacology University of Miami Miller School of Medicine, Miami, FL 33136;

^3^ Department of Physiology and Biomedical Engineering, Mayo Clinic Rochester, Rochester, MN 55905

Myosin motility velocity vs bulk concentration

**
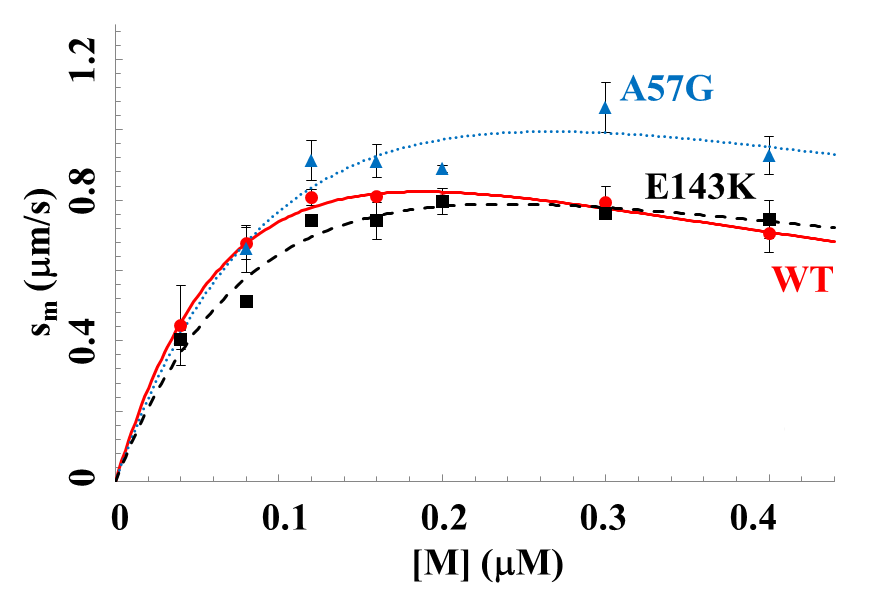
**

**Figure S1**. In vitro motility velocity vs bulk myosin concentration for WT (red circles), A57G (blue triangles), and E143K (black squares) with error bars indicating standard deviation. Smooth lines are the sum of two fitted exponential curves to indicate trends. Motility velocity increases with increasing αmys bulk concentration until reaching maximum at 0.14-0.20 μM and then slightly decreasing.


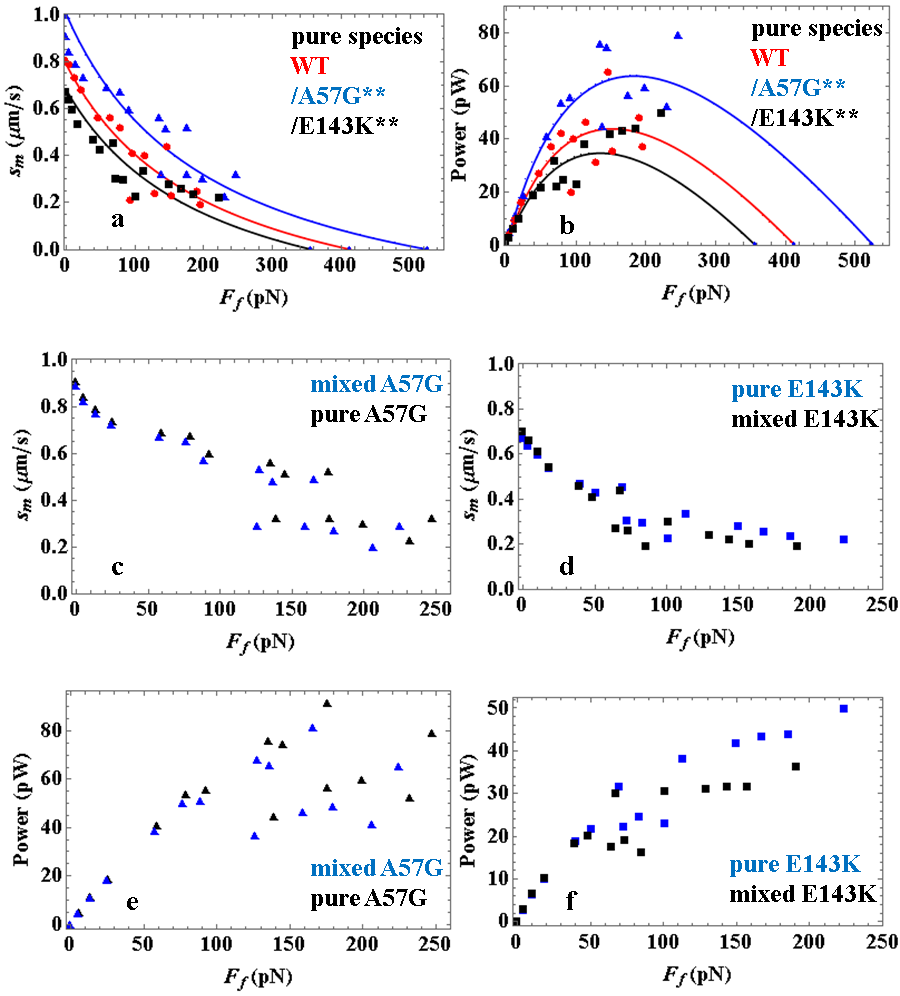
Effect of myosin mixtures on motility.

**Figure S2**. In vitro ensemble motility results from pure and mixed species of WT, A57G, and E143K. **Panels a & b** indicate pure species curves equivalent to the mixed species curves in **Figure 9 panels a & b**. WT curves are identical in **Figures S2 & 9**. **Panel b** shows that isometric forces are 357 or 526 pN for pure E143K or A57G, respectively, compared to 326 or 488 pN for the mixed species (**Table 1**). Pure and mixed species are compared pairwise in **panels c-f** showing their qualitative similarity. **Panels c & d** contain velocity vs drag force and **panels e & f** the power vs drag force.
